# Unexpected radiation pneumonitis after SIRT with significant decrease in DLCO with internal radiation exposure: a case report

**DOI:** 10.1186/s12880-020-00452-9

**Published:** 2020-05-19

**Authors:** Selin Kesim, Tunc Ones, Emel Eryuksel, Feyyaz Baltacioglu, Derya Tureli, Salih Ozguven, Tanju Yusuf Erdil

**Affiliations:** 1 Department of Nuclear Medicine, Marmara University Istanbul Pendik Education and Research Hospital, Fevzi Çakmak Mah. Muhsin Yazicioglu Cad. No:10 Ust Kaynarca / Pendik, 34899 Istanbul, Turkey; 2 Department of Pulmonary and Critical Care, Marmara University Istanbul Pendik Education and Research Hospital, Istanbul, Turkey; 3 Department of Radiology, Marmara University Istanbul Pendik Education and Research Hospital, Istanbul, Turkey

**Keywords:** Selective internal radiation therapy, Radiation induced pneumonitis, Diffusion capacity of the lungs for carbon monoxide

## Abstract

**Background:**

In the last years, Selective Internal Radiation Therapy (SIRT), using biocompatible Yttrium-90 (90Y) labeled microspheres have emerged for the treatment of malignant hepatic tumors. Unfortunately, a significant part of 90Y-labeled microspheres may shunt to the lungs after intraarterial injection. It can be predictable by infusing technetium-99 m-labeled macro-aggregated albumin particles through a catheter placed in the proper hepatic artery depending on the lobe to be treated with performing a quantitative lung scintigraphy. Radiation pneumonitis (RP) can occur 1 to 6 months after the therapy, which is a rare but severe complication of SIRT. Prompt timing of steroid treatment is important due to its high mortality rate. On the other hand, pulmonary diffusion capacity measured by carbon monoxide (DLCO) is an excellent way to measure the diffusing capacity because carbon monoxide is present in minimal amount in venous blood and binds to hemoglobin in the same manner as oxygen. Some authors reported that the most consistent changes after radiation therapy (RT) are recorded with this quantitative reproducible test. The relationship between the proportional reductions in DLCO and the severity of RP developing after this therapy may prove to be clinically significant.

**Case presentation:**

We herein present a patient who developed RP after SIRT that could be quantified using DLCO. To the best of our knowledge, this case is the first who developed unexpected RP after SIRT with significant decrease in DLCO with internal radiation exposure.

**Conclusions:**

RP is a very rare complication and may lead to a fatal outcome. Decline in DLCO could be a valuable parameter for follow-up and to identify potential candidates for RP and could be also another trigger for administration of steroid therapy with prompt timing in this patient group.

## Background

In last years, an alternative Selective Internal Radiation Therapy (SIRT), using Yttrium-90 (90Y) labeled microspheres which are infused directly into the hepatic arterial circulation have arisen for the treatment of malignant hepatic tumors. Unfortunately, a significant part of 90Y-labeled microspheres may shunt to the lungs after intraarterial injection. It can be predictable by simulating microsphere delivery and distribution by infusing [99 m] technetium-labeled (Tc-99 m) macro aggregated albumin (MAA) particles (5–50 μm in diameter) through a catheter placed in the right, left or proper hepatic artery depending on the lobe to be treated with performing a quantitative lung scintigraphy. The probability of radiation induced pneumonitis (RP) increases when > 15% of the 90Y-labeled microspheres is shunted into the lungs [1–3]. Therefore, SIRT is relatively contraindicated where there is a hepatopulmonary shunt which would lead to exposure of the lungs greater than 30 or 50 Gy, in a single treatment or in multiple sessions respectively [4].

The pulmonary diffusing capacity for a gas provides an estimate of the rate of transfer of that gas from the alveoli into capillary blood. Pulmonary diffusion capacity measured by carbon monoxide (DLCO) is an excellent way to measure the diffusing capacity because carbon monoxide is present in minimal amount in venous blood and binds to hemoglobin in the same manner as oxygen. Some authors reported that the most consistent changes after radiation therapy (RT) are recorded with this quantitative reproducible test [5–7].

Radiation pneumonitis (RP) can occur 1 to 6 months after the therapy, is a rare but severe complication of SIRT. Prompt timing of steroid treatment is important due to its high mortality rate [8]. Lin et al. reported the formation of interstitial pneumonia after SIRT in a patient who had a pulmonary shunt of 17% with the presence of resin microspheres in the histopathologic specimen in 1994 [2]. After this case report, Leung et al. stated the mortality rate of 60% in five patients who developed RP after the same treatment [3]. In 2015, another asymptomatic patient who was diagnosed with RP after SIRT as verified both by histopathologic examination and imaging findings was published by Dobrocky et al. [9].

There are some publications in the literature examining the relation between the reduction in DLCO and RP. Based on the theory that reduction in pulmonary function in patients with symptomatic RP can be measured by using DLCO, Guerra and colleagues researched the relationship between the change in DLCO and RP after external RT in 140 patients [10]. These researchers found that patients who had showed higher proportional reductions in DLCO were more likely to have high-grade RP. Additionally, in a recent study Ones et al. examined the effect of internal radiaton exposure of the lungs in patients undergoing SIRT using DLCO, which is a reliable indicator of lung function [11]. The most important difference between this work and others lies in the type of radiation exposure, which was internally administered in the former. Notably, in this study none of the patients developed RP during the follow-up and no changes in DLCO were found both after the first and second SIRT procedures [11].

The relationship between the proportional reductions in DLCO and the severity of RP developing after this therapy may prove to be clinically significant. We herein present a patient who developed RP after SIRT that could be quantified using DLCO.

## Case presentation

**Fig. 1:**
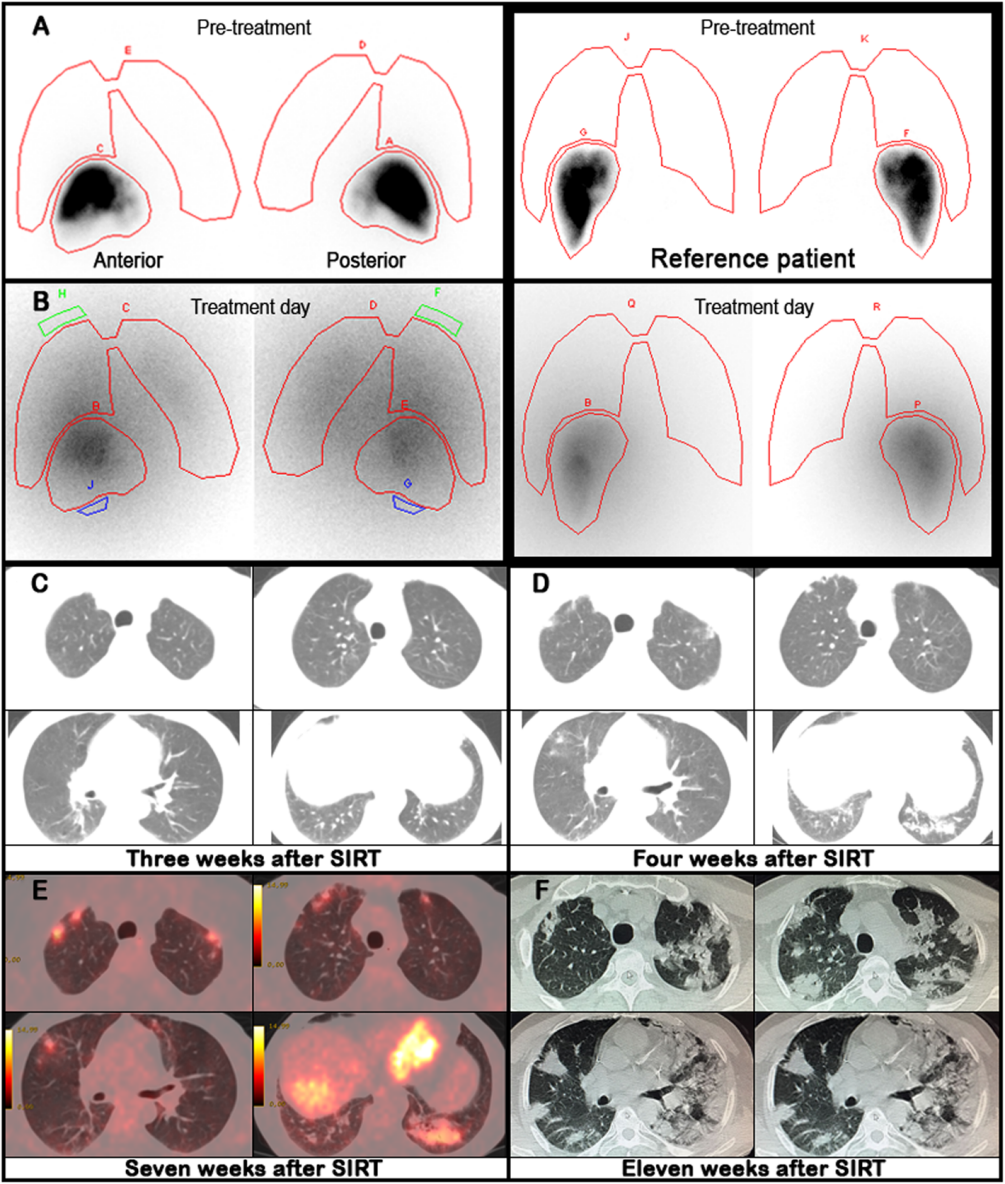
Comparison of the planar scintigraphic images of the case (**1a**, **1b**) and reference patient for liver-lung shunt imaging and transaxial fluorine-18 fluorodeoxyglucose positron emission tomography (18F-FDG PET/CT-** 1e **1E) / computed tomography (CT- **1c**, **1d**, **1f**) images obtained after selective internal radiation therapy (SIRT)

**Fig. 2:**
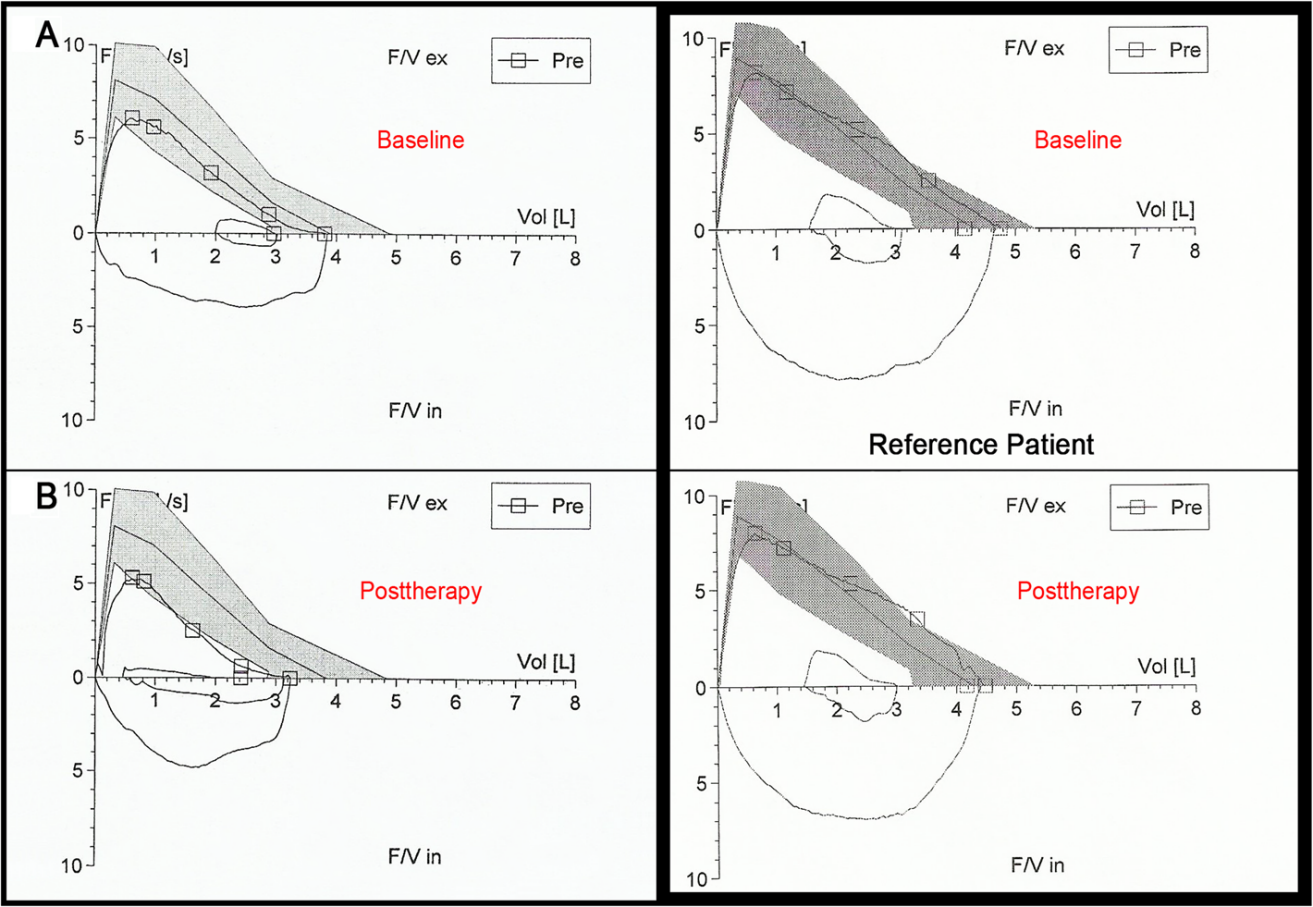
Comparison of lung diffusing capacity for carbon monoxide (DLCO) of the case (**2a**, **2b**) and reference patient in pre−/posttherapy

A 54-year-old man who was diagnosed with HBV-related hepatocellular carcinoma had successfully undergone left lateral segmentectomy for a lesion measuring 5 cm in maximum diameter. Four months after the surgery, whole-body 18-fluorine-fluorodeoxyglucose positron-emission-tomography/computed tomography (18F-FDG PET/CT) was performed due to the increased alpha fetoprotein (AFP) levels revealed malignant lesions mainly in the medial segment of the left hepatic lobe. The patient was not eligible for liver transplantation because of accompanying congestive heart failure. Additionally, due to the lesion’s proximity to the nearby vessels, radiofrequency ablation was not found to be appropriate. Stereotactic ablative radiation therapy is not available in our hospital and based on a multidisciplinary tumor board decision, the patient was referred to SIRT for the lesions in the left hepatic lobe. 99mTc-MAA particles were administered for liver-lung shunt calculation by planar imaging. It was demonstrated a lung shunt fraction (LSF) of 5% (Fig. 1a). Since there was no evidence that could interfere with the treatment, transarterial 32 mCi ^90^Y resin microspheres (SIR-Spheres, Sirtex Medical Limited, North Sydney, Australia) delivered via hepatic artery. Posttherapy bremsstrahlung planar images showed an unexpected liver-lung shunt (Fig. 1b). The patient was closely monitored by DLCO. The mean baseline DLCO was 98% of predicted value (Fig. 2a).

Three weeks after the SIRT, there was a significant decrease in DLCO (79% of predicted), while the patient was asymptomatic and the saturation of peripheral oxygen (SpO2) level was 99%. Chest radiogram and computed tomography (CT) of the thorax did not show any evidence of RP (Fig. 1c).

Four weeks after the SIRT, DLCO reduction was more remarkable (77% of predicted - Fig. 2b) and the SpO2 level was 97%. CT scan was assessed for the presence, extent, and anatomic distribution of the areas with ground-glass attenuation in the pulmonary parenchyma (Fig. 1d). The patient had no fever or dyspnea, but reported dry cough. Therefore, the patient was hospitalized and intravenous prednisone given at 1 mg/Kg/day with antibiotic therapy.

Seven weeks after the SIRT, 18F-FDG PET/CT scan was demonstrated 18F-FDG uptake in the multiple ground-glass opacities in both lungs compatible with RP (Fig. 1e). The patient was rehospitalized and glucocorticoid treatment with modified antibiotherapy continued.

Eleven weeks after the SIRT, CT scan demonstrated bilateral diffuse, patchy ground-glass attenuation/consolidation areas seemed to be progressed (Fig. 1f). Meanwhile, SpO2 decreased from 92 to 82%. The patient was transferred to the intensive care unit, but one week after he died due to pulmonary complications.

## Discussion and conclusions

To the best of our knowledge, this case is the first who developed unexpected RP after SIRT with significant decrease in DLCO with internal radiation exposure.

The alveolar-capillary complex is the most radiosensitive subunit of the lung and after the radiation exposure, initial cytokine release occurs within 2 weeks with no symptoms [12]. The second phase of this condition is characterized by hypoxemia and lung hypoperfusion. This phase begins 4–6 weeks after the initial cytokine release [12]. Lung injury has also been notified in areas where not exposed to radiation in patients receiving external RT and CD4(+) lymphocytic alveolitis similar to hypersensitivity pneumonia was described regardless of radiation exposure [13]. The proportional reductions in DLCO is thought to mirror a limited reserve of gas exchange resulting from the potential toxicity of RT.

The incidence of RP may be different between internal and external beam radiotherapy. In a study including 46 patients who undergone endobronchial brachytherapy due to the airway obstruction by non-operable malignant obstructive endobronchial lesions, RP developed only in those who were treated with external beam radiotherapy [14]. In an another paper, Leung et al. reported that only five of 80 patients developed RP who received SIRT alone [3]. Salem et al. reported that none of the 58 subjects had clinical RP who were treated with higher exposure doses than 30 Gy [4]. Additionally, Das and collegues have also recently reported a study of 103 patients who had LSF > 15% and no patient had features of RP [15]. Dobrocky et al. published a case report without related DLCO values where an asymptomatic patient had RP as confirmed both by imaging modalities and histopathologic evaluation [9].

There are some reports in the literature researching the relationship between the reduction in DLCO and RP. Aforementioned in the introduction section, Guerra and colleagues researched the possible relation between the extent of change in DLCO after external RT and RP [10]. In another study with internal dose absorption of the lungs, none of the patients developed RP during the follow-up and no changes in DLCO were found [11]. The relationship between the proportional reductions in DLCO and the severity of RP that forms after SIRT may be clinically important. If the expected association between the measured percent reduction in DLCO and severity of RP proves to be valid, then DLCO monitoring may allow to identify possible candidates for RP. The detection of the reduction in DLCO may prove to have clinical utility in this patient group, especially when the high radiation exposure related to CT scans is taken into consideration as recommended in the literature [16].

Diagnosis of RP is challenging and clinically based on the presence of classic symptoms including cough, shortness of breath, wheezing, timing and history of RT or SIRT, imaging findings and exclusion of alternative causes. Radiologic imaging findings demonstrating consolidation and atelectasis with multiple ground glass opacities suggestive of RP may be seen within the treatment field. Ocasionally, similar radiological findings on the outside of the treatment area may be seen probably due to the immunological response [8]. The first steroid treatment for RP was performed by Bluestein and Roemer in 1953 [17]. Since than, the mainstay of therapy consists of a long course of high-dose corticosteroid therapy. Oral prednisone is commonly administered in doses of 40–60 mg/day (or 1 mg/kg daily), slowly tapered over 8 to 12 weeks. Symptoms typically resolve within days. However, relapse of symptoms occurs in the majority of the patients during the taper of corticosteroids who require recurrent or continous therapy with higher doses and a slower taper. Non-steroidal anti-inflammatory drugs or inhaled steroids can be used for patients who have milder symptoms [8]. Other immunosuppressive agents such as azathioprine and cyclosporine can be prescribed for patients who are refractory to steroids or cannot tolerate steroids; however, experience is limited to case reports [8, 18, 19]. In the literature, treatment is only advised for symptomatic patients [8]. Standard steroid treatment protocol was not effective in this case for the relief of any symptoms related to RP and no improvement in radiological findings was seen. Additionally, post-SIRT bremsstrahlung planar imaging findings were unique, showing unexpected liver-lung shunt compared to our experience. Decline in DLCO could be a valuable parameter for follow-up and to identify potential candidates for RP and could be also another trigger for administration of steroid therapy with prompt timing in this patient group.

It should be remembered that RP is a very rare complication and may lead to a fatal outcome as radiation induced liver disease [20]. Prompt timing of steroid treatment is important in this clinical situation and closely monitoring the high-risk group with DLCO values could be more appropriate for this life-saving medical treatment. Further large studies to definitively test and better delineate DLCO changes after SIRT should be performed with different protocols.

## Data Availability

All data generated or analysed during this case report are included in the manuscript and its supplementary information files.

## Abbreviations

SIRT: Selective internal radiation therapy
RP: Radiation pneumonitis
DLCO: Diffusion capacity of the lungs for carbon monoxide
^90^Y: Yttrium-90
Tc-99 m: Technetium-99 m
MAA: Macro-aggregated albumin
RT: Radiotherapy
18F-FDG PET/CT: 18-fluorine-fluorodeoxyglucose positron-emission-tomography/computed tomography
AFP: Alpha fetoprotein
LSF: Lung shunt fraction
CT: Computed tomography
